# Human papillomavirus 16/18 seroprevalence in unvaccinated women over 30 years with normal cytology and with high grade cervical abnormalities in Australia: results from an observational study

**DOI:** 10.1186/s12879-014-0676-z

**Published:** 2014-12-21

**Authors:** Louiza S Velentzis, Freddy Sitas, Dianne L O’Connell, Jessica Darlington-Brown, Sam Egger, Rohit Sinha, Emily Banks, Ian H Frazer, Karen Canfell

**Affiliations:** Cancer Research Division, Cancer Council NSW, King’s Cross, 1340 NSW Australia; The University of Queensland Diamantina Institute, Brisbane, 4102 QLD Australia; National Centre for Epidemiology and Population Health, Australian National University, Canberra, 0200 ACT Australia; Lowy Cancer Research Centre, Prince of Wales Clinical School, University of New South Wales, NSW Sydney, 2052 NSW Australia

**Keywords:** HPV 16, HPV18, Seroprevalence, High grade cervical dysplasia, Age

## Abstract

**Background:**

Australia commenced human papillomavirus (HPV) vaccination in 2007, with a two-year catch-up to the age of 26; catch-up cohorts are thus now entering their thirties. Plans for monitoring vaccine impact involve pre- and post-vaccination assessment of cervical HPV DNA in the general population and in high grade abnormalities. Although HPV serology is less sensitive than DNA genotyping, it assesses lifetime exposure and may be easier to measure in the general population. However, benchmark pre-vaccination seroprevalence of vaccine-included types in unvaccinated women with high grade abnormalities has not previously been reported.

**Methods:**

We assessed seroprevalence for HPV16/18 from a population-based sample of 3,729 women with normal cytology and 971 women with confirmed high grade abnormalities (CIN2/3), aged 30–64 years, unvaccinated, and recruited in New South Wales in 2006–2010. We examined the variation in HPV16/18 seropositivity by age and in relation to a range of reproductive and behavioural characteristics in the subgroup of normal cytology women with no recent history of high grade cervical disease.

**Results:**

The HPV 16, 18 and combined seroprevalence was 19%, 7% and 24% among women with normal cytology, and 39%, 13% and 44% among women with CIN2/3, respectively. For both groups, HPV16/18 seroprevalence was highest at age 30–39 years and decreased with age. In multivariable analysis for women with normal cytology, HPV16 and HPV18 seropositivity were each associated with the number of lifetime sexual partners (p-trend <0.001 and 0.052, respectively) and for HPV16 this was also associated with age (p-trend <0.001) and prior diagnosis of Chlamydia (adjusted OR 1.89, 95% CI 1.27-2.80).

**Conclusions:**

The findings of this study inform pre-vaccination estimates of HPV seropositivity in women with normal cytology and women with high grade abnormalities. Almost a quarter of unvaccinated women aged over 30 years with normal cytology, and more than 40% of those with CIN2/3, had seroconverted to HPV 16 or 18. These findings provide a potential additional benchmark for assessing the effects of HPV vaccination.

**Electronic supplementary material:**

The online version of this article (doi:10.1186/s12879-014-0676-z) contains supplementary material, which is available to authorized users.

## Background

Human papillomavirus (HPV) types 16 and 18 are the most frequent oncogenic HPV types detected in invasive cervical cancers and precancerous cervical intraepithelial neoplasia grade 3 (CIN3) worldwide [1]. Australia was one of the first countries internationally to initiate a publicly-funded National HPV Vaccination Program, which commenced in April 2007. The program involves the quadrivalent vaccine (Gardasil™, Merck, Whitehouse Station, NJ, USA) which protects against infection with the oncogenic types HPV 16 and 18, and also types 6 and 11, which are implicated in the development of the majority of anogenital warts. The program involves routine vaccination of 12–13 year old girls at school, and until 2009 also involved a school, General Practitioners and community “catch-up phase” for women aged up to 26 years. Three-dose coverage rates in the first years of the program varied from 73% in 12–13 year old girls to ~30% in 20–26 year old women [2]. In 2013, the program was extended to boys. Early indications of the effects of the National HPV Vaccination Program have already been observed in vaccinated young cohorts– these include a decline in the prevalence of cervical HPV DNA for vaccine-included types in women aged 18–24 years [3], substantial declines in the incidence and prevalence of high-grade abnormalities in women up to 25 years old [4],[5] and a reduction in the risk of high grade cervical abnormalities in women who completed the vaccine series at the ages of 11–27 [6]. At present, as initial vaccination cohorts are entering their thirties, the effects of vaccination in women over 30 years old can now be evaluated.

HPV surveillance is an important mechanism of monitoring HPV prevalence at the population level, to assess the potential effects of cross-protection against non-vaccine-included types, and to exclude potential type-replacement of the vaccine-included types. HPV genotyping of normal cervical smears and for those with high grade abnormalities pre- and post-vaccination, has been proposed as part of such a program in Australia [7] and more generally [8], to be able to monitor vaccine effectiveness between these separate groups. An additional or alternative (if resources are limited) monitoring tool to genotyping would be HPV seroprevalence. The presence of HPV DNA is a sensitive measure of current infection and approximately 90% of cervical HPV infections are cleared after two years of observation [9]. There is evidence that antibody levels to HPV infection are stable over more than 4 years of follow-up [10] and can be used as markers of past infection. However, only 54-70% [11]-[13] of women infected with HPV-16/18 are thought to seroconvert after natural infection and therefore serotyping is less sensitive than genotyping. Blood samples for serology can be obtained more readily and from a wide range of population groups (for example as residual specimens collected for other purposes) and thus serology offers logistical advantages compared to the collection of cervical samples for genotyping. Using repeat cross-sectional surveys, before and after vaccination, serology has the potential to complement other methods in monitoring vaccination impact.

For serology-based surveillance, benchmark data on seroprevalence in unvaccinated women are required. Large studies assessing age-stratified, pre-vaccination seroprevalence data in women with and without cervical dysplasia are sparse. In Australia, we identified only one study [14], using a convenience sample of men and women aged up to 69 years which was limited by lack of information on cytology/histological status and on potential risk factors for HPV exposure, which might be important for monitoring outcomes in higher risk groups. More generally, there are only a few large studies internationally that have examined factors associated with HPV16 and 18 seropositivity [15],[16], none of which were conducted in Australia. Therefore the aims of the current analysis were (1) to quantify HPV16, 18 and combined 16/18 seroprevalence by age among unvaccinated women over 30 years of age with a normal cytology smear or with histologically-confirmed CIN 2/3 (the ‘pre-vaccination benchmark’ data); and (2) to investigate the relationship between detection of HPV16 or 18 seropositivity and a range of sexual and behavioural characteristics.

## Methods

### Study design and recruitment

Data and samples for this analysis have been obtained from the Cervical Health Study, a large population-based case–control study conducted in New South Wales (NSW), which was designed primarily to assess the role of exogenous hormonal co-factors in the development of cervical pre-cancerous lesions. Recruitment was conducted between December 2006 and July 2011 in women aged 20–64 years, via invitation letters issued from the centralised NSW Pap Test Register (PTR) which records all cervical cytology and histology test results, as well as past disease, except for those associated with <1% of women who opt-out. General Practices were first approached to consent for invitations to be sent to women under their care. Invitation letters were then issued to eligible women with a new record of a cytological/histological test. Cases had a new registry record of a low or high grade precancerous cytological or histological abnormality while controls had a new record of a normal cytological result in the same week as the abnormal smear for the matched case. Screening histories for up to 5 years prior to study participation were obtained from the PTR. Following informed consent, participants completed a self-administered questionnaire assessing demographic and medical details, and lifestyle factors. In the first phase of the study (to 2010) participants optionally provided a blood sample (women aged 30–64 years only). The Cervical Health Study commenced prior to the vaccination catch-up program implementation (covering ages of 12–26 years) but continued throughout the duration of the program. Therefore, vaccination status was confirmed by self-report.

For the current analysis, HPV16/18 seropositivity was investigated in women with histologically confirmed CIN2/3 and in women with normal cytology. Data has been obtained from the Cervical Health Study, however, analyses were restricted to women aged 30 years of age and above, who provided both a questionnaire and a blood sample. Women who indicated in their questionnaire that they had been vaccinated against HPV were excluded (n = 4). Analyses were conducted on 4700 women including 3,729 women with normal cytology and 971 with histologically confirmed CIN2/3.

### Sample processing and serological evaluation

Blood samples were collected by phlebotomists from December 2006 to August 2010 at several laboratories in NSW. Blood was collected in serum separating tubes, left to clot at room temperature for 30 mins, centrifuged, aliquoted into 2 ml aliquots within 48 hours of collection and stored at −80°C until analysis. Serum samples were pre-randomised by study number prior to aliquoting to ELISA plates. 210 samples were analysed in duplicate. Samples were tested ‘blind’ at the University of Queensland for circulating antibodies to the HPV16/18 late capsid protein L1 using a HPV-neutralising assay based on pseudoviruses carrying a secreted alkaline phosphatase reporter gene as described by Pastrana et al. [17]. A sample with an optical density reading below 50% of the negative control was considered HPV seropositive. Separate assays were conducted for HPV16 and 18. Blinded repeat testing was conducted for a random 7% of samples.

### Data analysis

The seroprevalences of HPV16, HPV18 and HPV16/18 combined, in the two groups of women were estimated and 95% confidence intervals (CIs) were calculated using the exact binomial method. Subjects were categorised into age groups (30–39, 40–49, 50–59 and 60–64 years).

Factors associated with seropositivity to HPV16 only or HPV18 only were investigated in a subset of 3,462 of the control women with an index negative smear and a history of normal cytology in the last five years. To evaluate the characteristics associated with each HPV type separately, women (N = 267) who were seropositive for both HPV types were excluded from these analyses. In doing so we followed the same analytical strategy as a major prior National Cancer Institute (NCI) USA study [12]. As in the NCI study, we restricted our analysis to women who were positive only for the single HPV type of interest. Similar analysis was not conducted in women with CIN2/3, because separate risk factors exist for progression of established HPV infection to high grade disease, which include use of oral contraceptives, current use of tobacco, parity and age at first term pregnancy [18]-[21], making the interpretation of any associations between these risk factors and HPV seropositivity in women with confirmed high grade disease complex. Unconditional logistic regression was used to estimate odds ratios (ORs) and 95% CIs for each potential factor adjusting for the following variables; age group, smoking, number of children, age at sexual debut, number of partners in the last 5 years, number of partners before the last 5 years, number of lifetime partners, and diagnosis of Chlamydia trachomatis (C.*trachomatis*). Associations between seropositivity and use of hormone contraceptives or use hormone replacement therapy were not assessed because the numbers of HPV16 or HPV18 seropositive users (never, past and current) were too low to provide sufficient statistical power for sub-group analysis to be conducted. A complete case approach was adopted for the analysis. Dose response associations (p-trend) were examined for ordinal variables which could be treated as continuous assuming a linear trend in the models and p-nominal values were estimated for all variables. All analyses were conducted using SAS software, version 9.3.

### Ethical approval

The study was approved in 2004 by the Cancer Institute NSW Population Ethics Committee; reference number Ref 2004/05/073.

## Results

### HPV 16 and 18 seroprevalence

**Figure 1:**
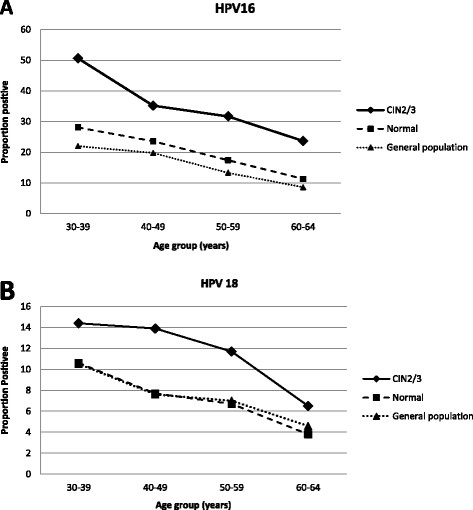
**Seroprevalence of (A) HPV16 and (B) HPV18, in women with CIN 2/3 and women with normal cytology, by age group.** [Data from Newall et al. [14] showing Australian, female population seroprevalence are also shown for comparison].

The overall HPV 16, 18 and combined HPV16 or 18 seroprevalence was 19.4% (723/3,729), 7.1% (266/3,729) and 23.5% (875/3,729) among women with normal cytology, and 38.5% (374/971), 12.7% (123/971) and 43.8% (425/971) among women with CIN2/3, respectively. Table 1 and Figure 1 present the seroprevalence results by age group. In both groups and for both HPV16 and HPV18, prevalence decreased with age; in women aged 30–39 years with normal cytology the combined seroprevalence was 33% and in women in the same age group with CIN2/3, it was over 55% (Table 1).

**Table 1:** **Seroprevalence of HPV16 and 18 in women with CIN 2/3 and women with normal cytology, by age group**

Age, years	CIN2/3 (N ^o^)	Normal cytology (N ^o^)	HPV16 seroprevalence % (95% CI)		HPV18 seroprevalence % (95% CI)		HPV16 or 18 seroprevalence % (95% CI)	
			CIN 2/3	Normal cyto ^a^	CIN 2/3	Normal cyto ^a^	CIN 2/3	Normal cyto ^a^
all	971	3729						
30-39	348	708	50.6 (45.2-55.9)	28.1 (24.8-31.6)	14.4 (10.9-18.5)	10.6 (8.4-13.1)	55.2 (49.8-60.5)	32.6 (29.2-36.2)
40-49	230	517	35.2 (29.0-41.8)	23.6 (20.0-27.5)	13.9 (9.7-19.1)	7.7 (5.6-10.4)	42.6 (36.1-49.3)	27.7 (23.8-31.7)
50-59	300	1945	31.7 (26.4-37.3)	17.4 (8.8-14.2)	11.7 (8.3-15.8)	6.7 (5.6-7.9)	36.3 (42.1-60.9)	21.8 (20.0-23.7)
60-64	93	559	23.7 (15.5-33.6)	11.3 (8.8-14.2)	6.5 (2.4-13.5)	3.8 (2.3-5.7)	28.0 (19.1-38.2)	13.6 (10.9-16.7)

^a^Cyto: cytology.

### Factors associated with HPV16 seropositivity in women with normal cytology

Table 2 presents the odds ratios for factors potentially associated with seropositivity. HPV16 seropositivity was significantly associated with younger age (p-trend <0.001); in the multivariable model, the odds of detecting anti-HPV16 antibodies in women 60–64 years of age was close to half that of women 30–39 years of age (OR 0.53, 95% CI: 0.36-0.78). Seropositivity was also associated with the number of lifetime partners (p-trend < 0.001). The odds of detecting antibodies against HPV16 were over six-fold higher in women with three or more sexual partners in their lifetime than women with only one partner (OR 6.50, 95% CI: 4.53-9.34), in the fully adjusted model. Seropositivity was also associated with the number of partners before the last 5 years (p-trend <0.001). In addition, ever having a diagnosis of C.*trachomatis* was associated with an increased risk of being HPV16 seropositive (OR 1.89, 95% CI: 1.27-2.80). After multivariable adjustment no other significant associations were seen.

**Table 2:** **Odds ratios for HPV16 seropositivity in women with normal cytology**

Characteristic	Anti-HPV16 Atb ^a^		Unadjusted OR (95% CI)	OR1 ^b^(95% CI)	OR2 ^c^(95% CI)	p-trend/p-nominal ^d^
	-ve	+ve				
**All women**	2659	522				
**Age**						
30-39	410	122	Ref.	n/a	Ref.	
40-49	345	93	0.90 (0.66-1.22)	n/a	0.93 (0.67-1.28)	<0.001
50-59	1469	259	0.59 (0.46-0.75)	n/a	0.71 (0.54-0.92)	0.003
60-64	435	48	0.37 (0.26-0.53)	n/a	0.53 (0.36-0.78)	
**Smoking**						
Never	1626	241	Ref.	Ref.	Ref.	
Past	781	206	1.77 (1.44-2.18)	1.76 (1.43-2.16)	1.18 (0.95-1.48)	n/a^e^
Current	252	75	2.00 (1.49-2.68)	1.91 (1.43-2.57)	1.16 (0.85-1.60)	0.310
**N** ^**o**^ **of children**						
0	245	81	Ref.	Ref.	Ref.	
1	296	81	0.83 (0.58-1.18)	0.82 (0.58-1.17)	0.91 (0.63-1.31)	0.146
2	1087	204	0.57 (0.43-0.76)	0.60 (0.45-0.81)	0.81 (0.59-1.10)	0.823
≥3	1031	156	0.46 (0.34-0.62)	0.51 (0.38-0.70)	0.78 (0.56-1.07)	
**Age at sexual debut**						
≥18	1609	255	Ref.	Ref.	Ref.	
17	392	94	1.51 (1.16-1.96)	1.44 (1.11-1.87)	0.95 (0.72-1.26)	
16	370	82	1.40 (1.06-1.84)	1.29 (0.98-1.70)	0.79 (0.59-1.07)	0.315
15	132	45	2.15 (1.50-3.09)	1.89 (1.31-2.74)	1.06 (0.71-1.57)	0.408
<15	84	32	2.40 (1.57-3.69)	2.13 (1.38-3.29)	1.00 (0.63-1.58)	
Prefer not to answer	72	14	1.23 (0.68-2.21)	1.24 (0.69-2.25)	0.56 (0.29-1.12)	
**C. trachomatis diagnosis**						
No	2545	467	Ref.	Ref.	Ref.	
Yes	87	46	2.88 (1.99-4.17)	2.73 (1.88-3.97)	1.89 (1.27-2.80)	n/a^e^
Prefer not to answer	27	9	1.81 (0.85-3.88)	1.73 (0.80-3.72)	1.21 (0.55-2.66)	0.006
**Partners in last 5y**						
0-1	2457	427	Ref.	Ref.	Ref.	
2	86	48	3.20 (2.22-4.63)	2.93 (2.02-4.25)	2.18 (1.48-3.21)	0.165
≥3	76	39	2.95 (1.98-4.39)	2.47 (1.64-3.71)	1.49 (0.97-2.29)	<0.001
Prefer not to answer	40	8	1.15 (0.53-2.47)	1.23 (0.57-2.65)	0.77 (0.33-1.80)	
**Partners before the last 5y**						
0-1	1240	74	Ref.	Ref.	Ref.	
2	249	45	3.12 (2.11-4.62)	2.99 (2.01-4.44)	2.92 (1.96-4.36)	<0.001
≥3	934	325	5.89 (4.51-7.69)	5.45 (4.17-7.13)	4.65 (3.48-6.22)	<0.001
Prefer not to answer	236	78	5.79 (4.07-8.23)	5.47 (3.86-7.76)	5.88 (3.95-8.76)	
**Lifetime N** ^**o**^ **sex partners**						
1	934	38	Ref.	Ref.	Ref.	
2	337	35	2.55 (1.59-4.11)	2.46 (1.52-3.96)	2.35 (1.45-3.80)	<0.001
≥3	1166	374	7.89 (5.59-11.14)	7.30 (5.16-10.33)	6.50 (4.53-9.34)	<0.001
Prefer not to answer	222	75	8.34 (5.50-12.65)	8.08 (5.32-12.27)	8.08 (5.14-12.69)	

^a^Ant: antibodies;

^b^ORs are adjusted for age only.

^c^ORs are adjusted for age, smoking, N^o^ of children, age at sexual debut, C.trachomatis diagnosis, partners in the last 5 years and partners before the last 5 years with the exception of the OR for lifetime N^o^ sex partners which were not adjusted for partners in last 5 years or partners before the last 5 years due to multi-collinearity between these three variables.

^d^p-values are for OR2.

^e^n/a: not applicable.

### Factors associated with HPV18 seropositivity in women with normal cytology

Results for HPV18 are presented in Table 3. Having an increasing number of sexual partners in a lifetime was associated with an increased risk of being HPV18 seropositive (p-trend = 0.052; p-nominal <0.001), and women with 3 or more lifetime partners had 6-fold increased odds (OR 6.16; 95% CI: 2.89-13.13) in comparison to women with one lifetime partner. Also, seropositivity was associated with the number of partners before the last 5 years (p-trend = 0.032). No other associations were observed in the fully adjusted analysis.

**Table 3:** **Odds ratios for HPV18 seropositivity in women with normal cytology**

Characteristic	Anti-HPV18 Atb ^a^		Unadjusted OR (95% CI)	OR1 ^b^(95% CI)	OR2 ^c^(95% CI)	p-trend/p-nominal ^d^
	-ve	+ve				
**All women**	3053	128				
**Age**						
30-39	510	22	Ref.	n/a	Ref.	
40-49	421	17	0.94 (0.49-1.79)	n/a	0.92 (0.48-1.78)	0.893
50-59	1651	77	1.08 (0.67-1.79)	n/a	1.23 (0.74-2.03)	0.378
60-64	471	12	0.59 (0.29-1.21)	n/a	0.76 (0.36-1.59)	
**Smoking**						
Never	1809	58	Ref.	Ref.	Ref.	
Past	928	59	1.98 (1.37-2.88)	1.99 (1.37-2.89)	1.40 (0.94-2.07)	n/a^e^
Current	316	11	1.09 (0.56-2.09)	1.07 (0.56-2.07)	0.70 (0.36-1.39)	0.065
**N** ^**o**^ **of children**						
0	311	15	Ref.	Ref.	Ref.	
1	359	18	1.04 (0.51-2.10)	1.06 (0.52-2.14)	1.13 (0.55-2.34)	0.352
2	1229	62	1.04 (0.59-1.86)	1.06 (0.59-1.89)	1.39 (0.75-2.55)	0.174
≥3	1154	33	0.59 (0.32-1.10)	0.60 (0.32-1.13)	0.86 (0.44-1.66)	
**Age at sexual debut**						
≥18	1800	64	Ref.	Ref.	Ref.	
17	463	23	1.40 (0.86-2.27)	1.35 (0.83-2.20)	0.99 (0.60-1.64)	0.941
16	428	24	1.58 (0.97-2.55)	1.53 (0.94-2.48)	1.02 (0.61-1.71)	0.605
15	171	6	0.99 (0.42-2.31)	0.96 (0.41-2.25)	0.63 (0.26-1.52)	
<15	108	8	2.08 (0.97-4.46)	2.02 (0.94-4.34)	1.25 (0.56-2.78)	
Prefer not to answer	83	3	1.02 (0.31-3.30)	1.01 (0.31-3.28)	0.39 (0.11-1.42)	
**C. trachomatis diagnosis**						
No	2898	114	Ref.	Ref.	Ref.	
Yes	123	10	2.07 (1.06-4.05)	2.02 (1.03-3.97)	1.58 (0.79-3.17)	n/a^e^
Prefer not to answer	32	4	3.18 (1.11-9.14)	3.15 (1.09-9.09)	2.16 (0.71-6.55)	0.187
**Partners in last 5y**						
0-1	2770	114	Ref.	Ref.	Ref.	
2	129	5	0.94 (0.38-2.35)	0.92 (0.37-2.29)	0.73 (0.29-1.85)	0.939
≥3	107	8	1.82 (0.86-3.82)	1.83 (0.86-3.92)	1.34 (0.61-2.95)	0.499
Prefer not to answer	47	1	0.52 (0.07-3.78)	0.52 (0.07-3.82)	0.30 (0.04-2.36)	
**Partners before the last 5y**						
0-1	1295	19	Ref.	Ref.	Ref.	
2	280	14	3.42 (1.70-6.91)	3.32 (1.64-6.70)	3.14 (1.55-6.39)	0.032
≥3	1189	70	4.07 (2.44-6.80)	3.95 (2.35-6.61)	3.49 (2.00-6.06)	<0.001
Prefer not to answer	289	25	6.11 (3.30-11.31)	5.83 (3.17-10.74)	6.98 (3.56-13.68)	
**Lifetime N** ^**o**^ **sex partners**						
1	964	8	Ref.	Ref.	Ref.	
2	361	11	3.67 (1.46-9.20)	3.64 (1.45-9.14)	3.45 (1.37-8.65)	0.052
≥3	1455	85	7.02 (3.39-4.56)	6.92 (3.32-14.40)	6.16 (2.89-13.13)	<0.001
Prefer not to answer	273	24	10.63 (4.72-23.92)	10.43 (4.63-23.50)	11.12 (4.72-26.17)	

^a^Ant: antibodies;

^b^ORs are adjusted for age only.

^c^ORs are adjusted for age, smoking, N^o^ of children, age at sexual debut, *C.trachomatis* diagnosis, partners in the last 5 years and partners before the last 5 years with the exception of the ORs for lifetime N^o^ sex partners which were not adjusted for partners in the last 5 years or partners before the last 5 years due to multi-collinearity between these three variables.

^d^p-values are for OR2

^e^n/a: not applicable

## Discussion

To our knowledge this is the first study to provide population-based benchmarking information on HPV16 and 18 serology in unvaccinated women with normal cytology and with high grade abnormalities in Australia. The seroprevalence measurements obtained in unvaccinated women in this study indicate high levels of exposure to HPV 16/18, most notably in women in their thirties. As expected, seroprevalence for both HPV types was higher in women with CIN 2/3 than those with normal cytology. The highest seroprevalence in both groups was observed in 30–39 year old women and it decreased with age. We also found that the number of lifetime sexual partners was associated with the detection of HPV16 and 18 antibodies in the subgroup of women with a history of normal cytology and that this association was due to past exposure. In this subgroup, younger age and ever having a diagnosis of C.*trachomatis* were significantly associated with HPV16 seropositivity.

Population cervical HPV DNA prevalence for oncogenic types tends to peak earlier than seroprevalence, with the DNA prevalence pattern reflecting a high risk of current infection around the time that young women first initiate sexual activity. Age-specific patterns of infection for whole-of-population cervical HPV DNA prevalence tend to be comparable but slightly higher than those for women in the population with normal cytology (i.e. excluding the relatively small group with a cytological prediction of an abnormality), and most studies of population DNA prevalence, including the IARC HPV surveys [22], report their findings for women with normal cytology. A systematic review of the worldwide literature has demonstrated that the highest oncogenic cervical HPV DNA prevalence is observed in women aged <25 years, with decreasing prevalence in older age groups in most geographical regions [23]. In a pre-vaccination Australian survey, the peak population cervical HPV16 or 18 DNA prevalence in both Indigenous and non-Indigenous women was observed in women under 21 years of age [24].

In terms of seroprevalence, our finding of decreasing HPV16 and 18 seroprevalence with increasing age in unvaccinated mid-adult and older women with normal cytology is consistent with other Australian data, although, studies done with different assays and at different laboratories complicate comparisons across studies. For the age groups for which comparative data were available, we observed a close correspondence between our findings and the general population findings of Newall et al. [14], which included females in their teens and twenties, and identified a peak seroprevalence of HPV16 and 18 in the 30–39 year old age group. Our findings are also broadly consistent with previously published data from other settings. A review of population seroprevalence in low-resource countries [22] reported a tendency of HPV16 and 18 seroprevalence to increase from young to middle age and then decline or stabilize thereafter. A global review of 117 studies [25] reported peaks in seroprevalence for HPV16 in women 25–40 years old and for HPV18 in women 35–55 years old. Taken together with other studies our findings are consistent with the probability that, prior to vaccination, the peak for both HPV16 and 18 seroprevalence in the general population occurred at an older age than for DNA prevalence for these types. These differences between the peak ages for current infections and for seroprevalence are likely to reflect, in part, the cumulative nature of seroprevalence as a measure of exposure – rates in women in their thirties may reflect exposure after initiation of sexual activities in their teenage years but also through their twenties.

The underlying reasons for declining seroprevalence of these HPV types at older ages are uncertain, but may include waning of naturally-acquired immunity over time and/or changes in the risk of HPV infection within successive birth cohorts over the past several decades. Waning anti-HPV16 antibody levels could in part explain our observation of a decreasing prevalence with age [15] although one study has suggested that HPV16 antibody titres do not decrease with age [26]. Changes in exposure in younger versus older birth cohorts seems a plausible alternate or additional explanation [26], since younger age at first sexual encounter and a higher number of sexual partners are observed in younger cohorts in many high resource countries [27].

We also examined risk factors for seropositivity in women with a history of normal cytology over the last five years, a subgroup less likely to be experiencing a current HPV infection (which may lead to abnormal cytology). The lifetime number of sexual partners and the number of past partners were associated with the detection of HPV16 and 18 antibodies. These findings are consistent with previous reports from other settings [12],[15],[28]-[30] and reflect cumulative past exposure to HPV, and possibly increased frequency and more sustained HPV exposure over time, which could favour seroconversion and thus antibody detection. We also observed an association between women reporting a past C. *trachomatis* infection and HPV16 seropositivity; having a previous C. *trachomatis* infection may be a marker of additional risk beyond that associated with the lifetime number of partners *per se*, which could occur, for example, if it acted as a marker for contact with higher risk partners. A similar association has been observed previously between herpes simplex virus-2 seroprevalence and HPV seropositivity [31]. No evidence has been identified in large scale pooled analyses of the worldwide data for a possible alternate explanation that sexually transmitted infections act as a co-factor in HPV persistence and/or progression. We also found that increasing age was associated with lower odds of detecting anti-HPV16 antibodies, but interestingly, the association was not confirmed for HPV18 antibodies. Taken together, these findings suggest that in our study, cohort effects impacting on the lifetime number of partners may be sufficient to explain the decreasing seroprevalence for HPV18, but not for HPV16, at older ages.

A number of studies have reported on pre-vaccination cervical HPV DNA prevalence in women with confirmed high grade abnormalities in various populations. Our recent study in New Zealand reported that the HPV16/18 DNA prevalence in women with CIN2/3, peaked among women 20–29 years of age and decreased with older age [32], indicating that high grade abnormalities in younger women in the pre-vaccination era were more likely to have been caused by the vaccine-included HPV types. Broadly similar observations for women with cervical intraepithelial neoplasia have been reported in other countries [33]-[36]. In contrast to the extensive literature for cervical HPV DNA studies, we identified few studies of oncogenic HPV seroprevalence in a population-based sample of women presenting with histologically-confirmed high grade abnormalities. A study in Costa Rica examined HPV seroprevalence in women with high grade abnormalities but participants were HPV16 or 18 DNA positive and thus the findings cannot be directly compared to the current study [12]. Among women with CIN2 recruited in a US trial, the peak seroprevalence for HPV16 and 18 was observed for the age group 20–25 years decreasing in older age groups [37], although the median age of participants was 27 years and thus limited comparative data are available for women aged over thirty years. Therefore, although directly comparable results from other studies are not available, our finding of an overall 44% HPV16/18 seroprevalence in women over 30 years old with high grade abnormalities is broadly consistent with studies showing that about half of all HPV-positive histologically confirmed high grade lesions are associated with the presence of cervical HPV16/18 DNA (as opposed to other oncogenic types), but this proportion decreases in women over 30 years of age [32],[38].

This study is subject to potential limitations. Participants were recruited from the NSW Pap Test Register and therefore results may not be representative of the general female population of NSW. Although few women opt-out of the register, recent national figures indicate that only 57% of women 20–69 years of age attended cervical screening [39]. Furthermore, due to the unavailability of cervical DNA samples, it was not possible to carry out HPV genotyping; it is therefore unknown whether study participants had prevalent HPV cervical infections of the same genotype. However, this study has a number of strengths including the relatively large sample size, the availability of risk factor and demographic information, the 5 year cervical screening history for participants, the concurrent assessment of HPV16 and 18 seropositivity in women with CIN 2/3 and women with normal cytology and recruitment from a single source.

## Conclusion

By providing information on seroprevalence of HPV types 16 and 18 in unvaccinated women with normal cytology and with high grade abnormalities, this study establishes the possibility of using serology for monitoring changes in population level exposure over time. However, as HPV seropositivity due to natural infection is based on repeated and persistent exposure rather than incident infection, serology results have to be interpreted with caution; furthermore vaccine exposure will lead to complex serological patterns for vaccine-included types in cohorts offered vaccination. We also note that when used in combination with individual-level information on vaccination status (which is available, in principle, for accredited research purposes, through Australia’s National HPV Vaccination Register), serology could be used to measure natural HPV 16 and 18 exposure in populations where vaccinated women mostly received the vaccine at a young enough age to be protected from HPV 16 and 18 infection. This is the expectation for Australia, in years to come, given that successive cohorts of 12–13 year old schoolgirls will receive the vaccine through the vaccination program and given that the government no longer subsidises the vaccination of older women. In such populations, vaccinated women can be reasonably assumed to be unexposed to natural infection with HPV 16 and 18 because of the protection provided by the vaccine. Serology may then have a role to estimate HPV 16/18 exposure in the unvaccinated remainder of the population.

## Authors’ original submitted files for images

Below are the links to the authors’ original submitted files for images. Authors’ original file for figure 1

## Abbreviations

HPV: Human Papilloma Virus
CIN2/3: Cervical intraepithelial neoplasia grades 2 and 3
