# Supplementary figures and images for: Human papillomavirus 16/18 seroprevalence in unvaccinated women over 30 years with normal cytology and with high grade cervical abnormalities in Australia: results from an observational study
Source: BMC Infect Dis. 2014 Dec 21;14:3861. doi: 10.1186/s12879-014-0676-z (PMC4299782; doi:10.1186/s12879-014-0676-z)

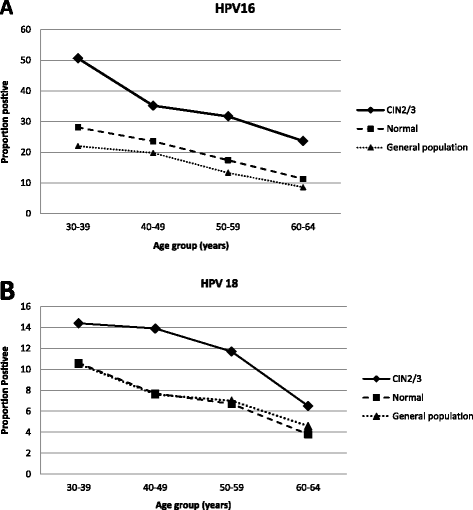

Supplement: Supplementary file 1 — Authors’ original file for figure 1 [file 12879_2014_676_MOESM1_ESM.gif]
